# Multiple Sclerosis Classification Using the Local Divergence Exponent: Parameters Selection for State-Space Reconstruction

**DOI:** 10.3390/s25092819

**Published:** 2025-04-30

**Authors:** L. Eduardo Cofré Lizama, Liuhua Peng, Tomas Kalincik, Mary P. Galea, Maya G. Panisset

**Affiliations:** 1 Department of Allied Health, School of Health Sciences, Swinburne University of Technology, Hawthorn, Melbourne, VIC 3122, Australia; 2 Department of Medicine, Royal Melbourne Hospital, The University of Melbourne, Melbourne, VIC 3052, Australia; m.galea@unimelb.edu.au (M.P.G.); mpanisset@unimelb.edu.au (M.G.P.); 3 School of Mathematics and Statistics, The University of Melbourne, Parkville, VIC 3050, Australia; liuhua.peng@unimelb.edu.au; 4 Neuroimmunology Centre, Department of Neurology, Royal Melbourne Hospital, Melbourne, VIC 3052, Australia; tomas.kalincik@unimelb.edu.au; 5 Clinical Outcomes Research Unit, The University of Melbourne, Melbourne, VIC 3052, Australia; 6 Australian Rehabilitation Research Centre, Royal Melbourne Hospital, Melbourne, VIC 3052, Australia

**Keywords:** stability, Lyapunov, dynamic, multiple sclerosis, state space

## Abstract

Background: Using the local divergence exponent (LDE), it has been concluded that walking stability is impaired in people with multiple sclerosis (pwMS). However, the use of several calculation approaches hinders comparisons across studies. We aimed to determine whether using different parameters for state space reconstruction to calculate LDE affects the classification of pwMS. Methods: A total of 55 pwMS and 23 controls walked up and down a 20 m corridor for 5 min. The LDE was calculated using three different combinations of n-dimensions (*d_E_*) and time delays (τ): (a) trial-specific, (b) median across subjects, and (c) fixed *d*_E_ = 5 and τ = 10. The LDE was calculated using vertical (VT), mediolateral (ML), and anteroposterior (AP) accelerations, the norm (N), and 3D data from sensors placed on the sternum and lumbar. Classification accuracy across results obtained with different parameter combinations was compared using a Quadratic Discriminant Analysis (QDA). Results: The best classification accuracy, 84%, was achieved when using the LDE obtained with norm acceleration data from the sternum sensor with a fixed *d_E_* = 5 and τ = 10 and considering speed as a covariate. Lumbar LDEs were less accurate than sternum LDEs. Conclusions: LDEs calculated with a fixed *d_E_* = 5 and τ = 10 for the norm acceleration from a sternum-placed sensor can best classify pwMS. Using fixed parameters for the state space reconstruction, and consequently LDE calculation, can simplify the implementation of the LDE as a mobility biomarker in MS and provides evidence for future consensus for its calculation.

## 1. Introduction

The local divergence exponent (LDE) has emerged as a valuable measure for quantifying walking stability in multiple sclerosis (MS) [1]. Several studies have consistently reported that stability in people with MS (pwMS) is impaired even at early stages of the disease [2,3,4,5]. This decline in stability has been further linked to central nervous system damage, suggesting a neural basis for the LDE changes in pwMS [6]. Identifying gait instability in MS is crucial, particularly in pwMS with no or minor gait impairments (e.g., EDSS < 4.0), as there remains a need for more sensitive measures to track early deterioration, disease progression, and ([non-]pharmacological) intervention effectiveness for which the LDE can be a sensitive biomarker [1,3].

Measuring stability with the LDE, however, requires several methodological choices that could significantly impact results [7]. These choices include the walking task performed, sensor data used (e.g., acceleration or angular velocity), data source (e.g., IMUs or motion capture), sensor location (e.g., sternum or lumbar), length of the dataset (number of strides), the algorithm used for LDE calculation (e.g., Rosenstein or Wolf), whether directional or 3D signals are analyzed, and state space reconstruction parameters (embedding dimension (*d_E_*) and time delay (τ)) [8,9,10,11]. Each of these decisions can influence the interpretation of walking stability in pwMS, making it difficult to compare studies and potentially leading to conflicting conclusions. Similar concerns have been raised in research using LDE to assess walking stability in older adults [7].

Regarding the type of data considered, studies using angular velocities have reported lower stability (higher LDE) in pwMS, with greater LDE values prospectively predicting fall risk [4,12]. However, most research has used linear accelerations, particularly from the sacrum/lumbar region, as these signals may better capture directional motion—the primary objective of walking. While previous studies have typically analyzed separate directional components (vertical [VT], mediolateral [ML], and anteroposterior [AP]), Janshen et al. introduced an alternative approach by using the norm (N) acceleration, which has not been widely explored in pwMS [13].

A consensus exists regarding data preprocessing, with most studies normalizing data to a fixed sample rate × n strides [11]. Similarly, the Rosenstein algorithm is the most frequently used method for calculating divergence, as it has been shown to outperform the Wolf method [14,15]. However, a critical yet underexplored methodological choice in the LDE calculation, particularly when used to determine gait stability in clinical populations, is state space reconstruction [9]. This requires selecting two key parameters: the embedding dimension (*d_E_*) and the time delay (τ) between dimensions [9,11,16]. Most studies calculate *d_E_* using the Global False Nearest Neighbors (GFNN) [17] method and τ using the Average Mutual Information (AMI) [17] method for each trial, after which the mean or median across all trials is used to reconstruct the state space and calculate the LDE [18]. Some studies, however, use fixed values for *d_E_* and τ based on the prior literature and skip GFNN and AMI calculations. Another approach for state space reconstruction is using the individual trial’s *d_E_* and τ, which has shown to be slightly more sensitive to differentiate between “known groups” [11]. This individualized method has not yet been applied to studies investigating walking in pwMS.

A recent study found that using fixed or individually calculated τ values did not affect LDE reliability in young adults walking on a treadmill, suggesting that methodological choices for τ may have limited impact [10]. However, other research suggests that fixed *d_E_* and τ values yield better test–retest reliability in healthy individuals [18]. Another study explored the effects of data length and preprocessing methods on *d_E_* and τ for the AP, ML, and VT directions, separately [9]. Overall, this study found that τ was not uniform, ranging from 4 to 16, and that *d_E_* reached a steady state only after 300 gait cycles. Unfortunately, achieving over 300 gait cycles in neurological populations does not seem feasible even when using data from a 6mWT. State space reconstruction is essential for calculating the LDE, as the selection of embedding dimension (*d_E_*) and time delay (τ) can alter the system’s topology and distort LDE values; however, no consensus exists on its application for pwMS or other clinical populations [10]. Taken together, choosing the right *d_E_* and τ parameters for state space reconstruction and consequent use of the LDE is not a trivial decision.

Given the growing interest in LDE as a potential disease progression marker and outcome measure in clinical trials, it is essential to establish how different state space reconstruction approaches influence, for example, the ability of the LDE to correctly classify pwMS. Standardizing methodological choices is key to improving comparability between studies and ensuring clinical utility of the LDE as a gait stability measure. Hence, the aim of this study was to determine how different parameter selections for state space reconstruction affect the classification of pwMS at early disease stages when using LDE to assess gait stability. In this study, we compared the effect of using different parameter selection methods: individual (per-trial), median across subjects, and fixed values of embedding dimension (*d_E_*) and time delay (τ). By clarifying the impact of these methodological choices, this study will contribute to the standardization of LDE as a stability metric for pwMS, enhancing its potential as a diagnostic and outcome measure for disease monitoring and clinical trials.

## 2. Materials and Methods

***Participants:*** A total of 55 people with relapsing–remitting multiple sclerosis (RRMS) and 23 healthy adults provided written informed consent. Inclusion criteria for pwMS were (a) <15 years since onset and (b) EDSS < 4.0. The latter criteria ensured inclusion of pwMS with no or minor gait impairments. Exclusion criteria for both groups included presence of (a) other neurological conditions, (b) cardiovascular disease, (c) orthopedic conditions, or (d) pregnancy or <5 months post-partum. This study was approved by the Human Research Ethics Committee at the Royal Melbourne Hospital (HREC2019.093) and informed consent was obtained from all participants prior to assessments.

**Figure 1:**
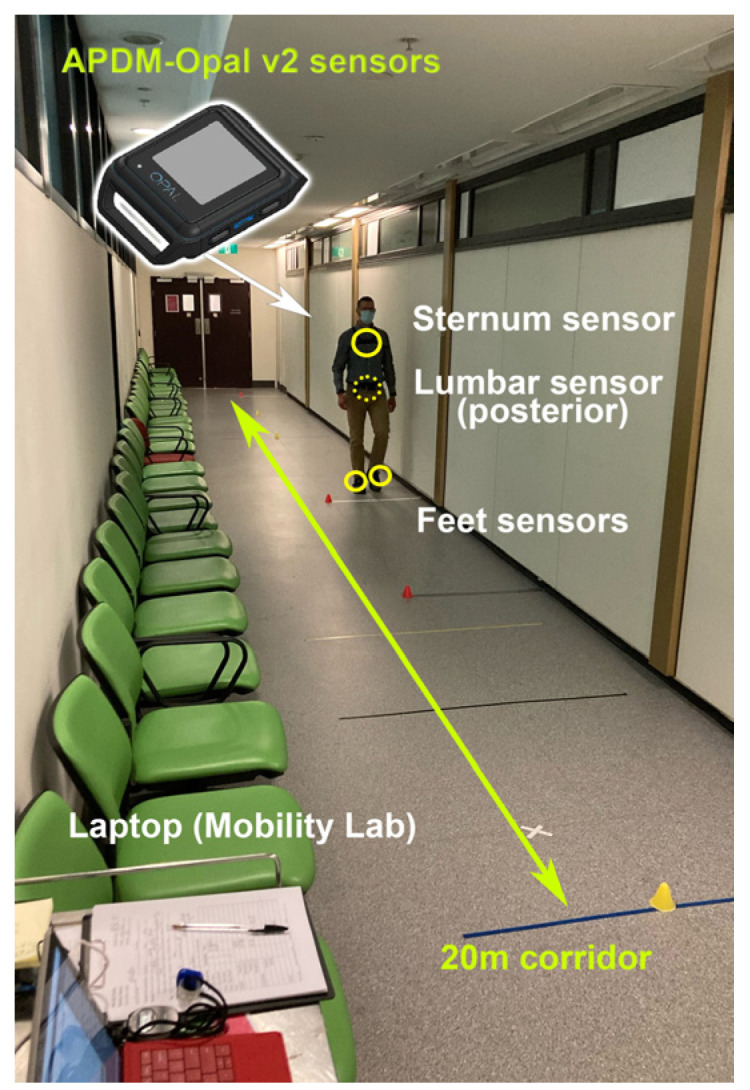
Experimental protocol presenting a patient walking along the 20 m hospital corridor where assessments were performed. APDM Opal v2 sensors locations are highlighted in yellow circles.

***Protocol:*** All participants were recruited from the Multiple Sclerosis and Neuroimmunology Clinic at the Royal Melbourne Hospital. They were instructed to walk continuously for 5 min along a 20 m unobstructed corridor located within the MS clinic. At the end of each lap, marked by a line on the floor, participants were asked to turn around and continue walking until the assessor instructed them to stop. Although participants were informed that they could stop at any time if they felt uncomfortable, fatigued, or unsafe for any reason, but no one chose to do so. Participants wore a system of four inertial measurement units (IMUs) that recorded 3D accelerations and angular velocities at 128 Hz (APDM-Opals v2 and Mobility Lab Software v2, Portland, OR, USA). The IMUs were securely attached to the lumbar spine (LUM), sternum (STR), and both feet using elastic straps (Figure 1).

**Figure 2:**
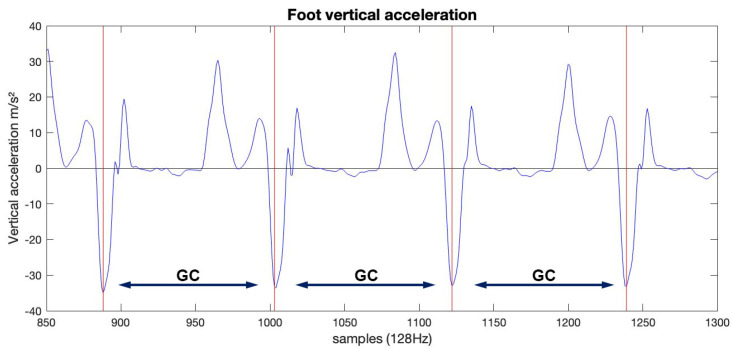
Vertical left foot acceleration (m/s^2^) time series in which local minimum (vertical red lines) identification was used to extract 150 gait cycles (GCs) across all laps.

***Data Analysis:*** This is a secondary analysis of an ongoing study for which some results are included in [3]. All data analyses were conducted in Matlab-R2024b (Natwick, MA, USA). Non-walking data (turns) were identified from peak angular velocity in the transverse plane; 1.5 s of (de)-acceleration data before and after the peak were removed. Gait cycles were identified using feet sensor data and a previously described algorithm in which gait cycles were extracted by identifying left foot sensor vertical acceleration local minima (Figure 2) [3]. Acceleration data for the middle 150 strides were extracted and used for further analyses [3,4]. For both LUM and STR sensors, three-dimensional (3D), and directional [vertical (VT), mediolateral (ML), anteroposterior (AP), and norm (N, Equation (1)] acceleration data were normalized to n strides ×100 samples (15,000 datapoints), separately [8,11]. For the state space reconstruction of the 3D data, we used two sets of fixed *d_E_* and τ values based on the previous literature: (1) *d_E_ =* 3 and τ *=* 10, and (2) *d_E_ =* 5 and τ *=* 10 [2,4,19,20]. To reconstruct the state space for the directional data (VT, ML, AP, and N), we used (1) each individual (*I*) subject’s trial-calculated *d_E_* (using the Global False Nearest Neighbors) [17] and τ, (using the Average Mutual Information) [21]; (2) the median (*M*) *d_E_* and τ across all subjects in each direction (VT, ML, AP, and N) and for each sensor (LUM and STR) separately; and (3) fixed (*F*) *d_E_ =* 5 and τ *=* 10 based on previous studies using the LDE in clinical populations (including pwMS). Median *d_E_* and τ values are presented in Table 1 The state space reconstruction is presented in Equation (2). Finally, we calculated the short-term LDE (0–0.5 strides) for each sensor (LUM and STR) and for each state space reconstructed (*I*, *M*, and *F*) utilizing the Rosenstein algorithm [14].

(1) $$N=\sqrt{a_{AP}^{2}+a_{ML}^{2}+a_{VT}^{2}}$$

where *N* is the norm;

*a* is acceleration in the AP, ML, and VT directions.

*S(t) = [z(t), z(t + τ), z(t +* 2*τ),…,z(t + (d_E_ − 1)τ)]* (2)

where *S*(*t*) is the reconstructed state space;

*z* is the selected one-dimensional time series;

*τ* is the time delay;

*d_E_* is the embedding dimension.

***Statistical Analysis:*** The dataset including 55 pwMS and 23 HCs was randomly split into training and testing sets with a 2:1 ratio. The Quadratic Discriminant Analysis (QDA) classifier was trained on the training set using LDE (short-term) under different combinations of sensor location, direction, and different choices of *d_E_* and τ [22]. We then calculated the classification accuracy on the test set and averaged the results over 1000 random splits. Previous studies suggest that better classification performance can be achieved when walking speed is taken into account. Since speed is also affected by aging and aging itself affects walking stability when measured with the LDE, we also performed a secondary analysis including both age and speed. QDA was performed using the original dataset without applying normalization or covariate adjustments. A mixed model was conducted to determine between-group comparisons in age, sex, height, and body mass index (BMI).

## 3. Results

### 3.1. Participant Demographics

Demographics for the HC and pwMS are presented in Table 2. Overall, pwMS (n = 55) had a median EDSS = 2.0. No significant differences in age, height, or sex ratio between HC and pwMS were found.

### 3.2. Classification Analysis

**Figure 3:**
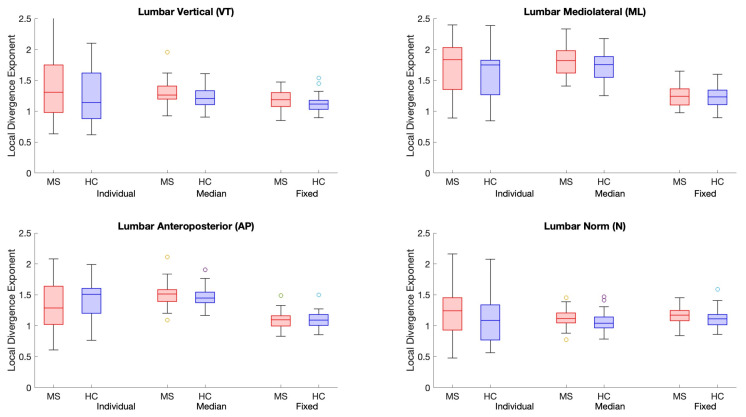
Boxplots present LDE values for the lumbar sensor LDEs calculated using individual, median, and fixed m and τ for the VT, ML, AP, and N time series. People with MS (pwMS) are presented in red, whereas healthy controls (HCs) are presented in blue.

**Figure 4:**
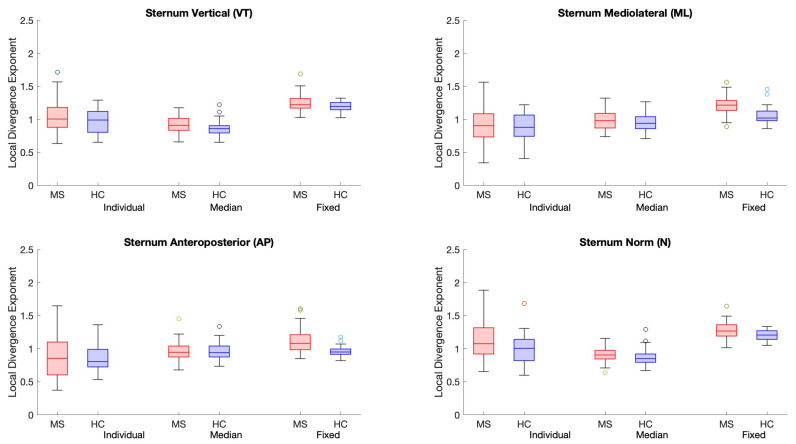
Boxplots present LDE values for the sternum sensor LDEs calculated using individual, median, and fixed m and τ for the VT, ML, AP, and N time series. People with MS (pwMS) are represented in red, whereas healthy controls (HCs) are represented in blue.

**Figure 5:**
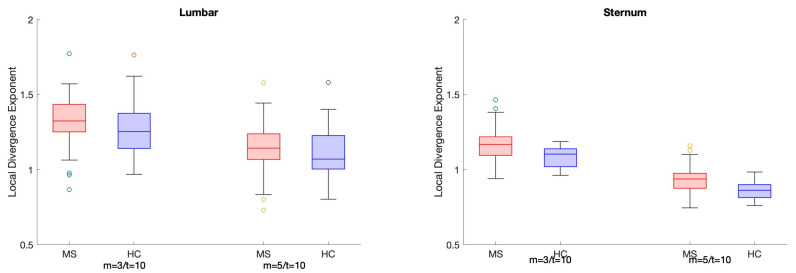
Boxplots present 3D LDE values for the lumbar and sternum sensors calculated using the 2 sets of fixed m and τ (*d_E_* = 3 and τ = 10, and *d_E_* = 5 and τ = 10). People with MS (pwMS) are represented in red, and healthy controls (HCs) are represented in blue.

Overall, classification performance of most calculated LDEs was improved when speed was controlled for (Table 3). Classification accuracy without considering speed is presented in Supplementary Table S1. Age marginally improved the classification accuracy of LDEs (Supplementary Table S2), whereas speed alone achieved 0.710 accuracy. Most sternum LDEs achieved accuracy >0.700, whereas the accuracy of lumbar LDE classification ranged between 0.679 and 0.717. The highest classification accuracy (0.826) was achieved when using the sternum’s norm (N) with a fixed *d_E_* = 5 and τ = 10. For the directional sternum LDEs, classification accuracy was the highest when using fixed *d_E_* and τ (>0.771). For the 3D LDEs, using both pairs of fixed *d_E_* and τ with sternum LDEs showed a better classification performance than lumbar. LDE values obtained using I, M, and F *d_E_* and τ for all time series (VT, ML, AP, and N) for the lumbar and sternum sensors as well as 3D LDEs are presented in Figure 3, Figure 4, and Figure 5, respectively.

## 4. Discussion

We aimed to compare the accuracy of classification of pwMS at early stages of the disease using the LDE calculated using different n-dimensions (*d_E_*) and delays (τ) for the state space reconstructions. Various parameter selection methods have been previously utilized in the literature. This hampers comparisons across studies to determine the clinical utility of the LDE as a supplementary diagnostic, for disease monitoring and progression, and as an outcome measure to assess intervention efficacy in pwMS [3]. The present study was motivated by the need to provide evidence towards achieving consensus in the standardization of LDE calculation, facilitating clinical implementation and enabling comparisons across studies. Understanding the consequences of parameter choices for state space reconstructions in calculating the LDE is crucial for using this as a classification tool and a mobility biomarker of disease progression in MS.

State space reconstruction is crucial for LDE calculation, as the choice of embedding dimension (*d_E_*) and time delay (τ) can alter the system’s topology and distort LDE values; however, no consensus exists on its application for pwMS or other clinical populations [10]. The optimal τ enables z(t) and z(t + τ) (Equation (2)) to represent system dynamics with minimal redundancy while preserving structure, resulting in an unfolded, informative state space [9]. In contrast, τ values that are too low or too high can distort LDE: low τ may suppress divergence or cause artificial convergence, while high τ may inflate LDE due to delayed copies no longer sharing information [23]. Similarly, a low dE may misidentify neighboring trajectories, while a high *d_E_* may overembed the attractor, resulting in a sparse structure and inflated LDE. Selecting appropriate τ and *d_E_* is therefore essential for calculating LDE from properly unfolded time series that reflect dynamic stability [14], particularly when LDE is used to quantify stability as a biomarker of gait deterioration and disease progression in neurological populations, such as pwMS.

We found that the best classification performance was achieved when using the norm acceleration (N) from the sternum with fixed parameters (*d_E_* = 5 and τ = 10). To our knowledge, N has only been used by Janshen et al. [13] who found that pwMS exhibited lower stability than HCs. However, that study differs from ours in several ways, including the use of treadmill walking, fixed *d_E_* = 3 and individual τ, lumbar sensor placement, and slope range, as well as using 0–0.25 of the delay vs. 0–0.5 of stride in our study. The use of sternum-derived LDEs has previously been shown to not only classify pwMS [3] but has also been associated with falls [24], clinical impairments [5], and fatigue [25].

Of note, most previous studies have used directional (VT, ML, and AP accelerations) or 3D time series [2,3,4,12,19,20,26,27], and most of them aimed to determine between-group differences (e.g., pwMS vs. HC). Only one previous study suggested that, although not the best model, using fixed parameters (*d_E_* = 3 and τ = 10) to calculate 3D LDE (considering speed) may help simplify the implementation of the LDE for the classification of pwMS [3]. Since N- and 3D-derived LDEs consider accelerations in all directions during walking, it is possible that inherent biological variability amongst pwMS can be better captured by these time series, hence improving classification accuracy [18].

The present study is one of the few to investigate the use of individual *d_E_* and τ to calculate LDEs in pwMS. Methodologically, this approach ensures the reconstructed attractor retains the same topological and dynamical properties as the original system. Pragmatically, the use of individually created state spaces allows for an immediate LDE calculation and report, which is relevant and preferable in clinical contexts. LDEs obtained with individually calculated *d_E_* and/or τ are slightly more sensitive to locomotion modes (walking and running at the same speed) than those obtained with mean parameter values [11]. However, van Schooten et al. found that individually calculated τ yielded lower reliability of the LDE than fixed and median LDE values in young adults walking overground [18].

Placing sensors in the lumbar area is often considered a proxy for the center of mass (CoM), the controlled variable in human motion [3]. Our findings, along with those of previous studies, suggest that the lower stability and higher classification accuracy observed in people with multiple sclerosis (pwMS) when using sternum-placed sensors may reflect the balance control system’s ability to prioritize CoM control to maintain walking stability—particularly in the early stages of the disease, when there are still available redundancies in the system. The increased instability observed in the upper trunk may be a consequence of the damaged central nervous system reallocating its resources. However, this hypothesis requires further investigation.

We performed four QDA classification analyses using (1) LDE, (2) LDE and speed, (3) LDE, speed, and age, and (4) speed alone (Supplementary Materials). Speed alone showed low classification accuracy; however, in combination it helped improve LDE’s classification accuracy. This may be because walking stability when measured with the LDE can be significantly affected by speed. The effect of speed can be avoided by using fixed speed treadmill walking; however, this may change the dynamic behavior of the system and not reflect daily-life walking [28,29]. It is noteworthy, however, that previous studies using fixed-speed treadmill walking have also found significant stability differences between pwMS and healthy controls [2,5,12,13,30]. What may pose, nonetheless, a difference in regard to the assessment conditions is walking in daily life, for which walking stability using the LDE has been shown to be significantly different to when in controlled environments [28].

Regarding the length of the walking dataset, this varies widely across studies in pwMS, ranging from as few as 7 strides to as many as 150 strides [2,25]. Previous research suggests that beyond 150 strides (*d_E_* = 5 and τ = 10), increases in statistical precision are minimal, making this an appropriate upper bound for clinical assessments [8,31]. However, Hussain et al. [9] showed that over 300 gait cycles are required to achieve a steady-state value of *d_E_* = 5 in all directions. This seems, however, impractical when assessing clinical populations such as pwMS, who may also struggle with fatigue issues [25]. Interestingly, in our group of pwMS for which we use 150 gait cycles, all calculated median number of dimensions were higher (*d_E_* = 7) than in studies exploring the impact of methodologies for the LDE calculation (*d_E_* = 5) in healthy young subjects [9,10,11,29,31]. Using healthy subjects’ *d_E_* to obtain the LDE in pwMS may therefore better serve the purpose of classification of patients with no evident gait impairments but may not allow full unfolding of the time series [5,26,27,30]. Further research should explore if using population-specific parameters may be better suited to monitoring progressive neurodegenerative diseases such as MS.

Our findings suggest that fixed *d_E_* = 5 and τ = 10, overall, can better classify pwMS at early stages of the disease. Fixed parameter values are often based on the previous literature assessing young healthy individuals which suggests that such values may better reveal subtle changes in walking dynamics in clinical populations. Two possible explanations for higher *d_E_* = 7 in pwMS are that either their motor behavior is more complex or perhaps there is more pathological noise in the motor control system. Considering that MS affects the central nervous system, and hence motor and balance control, the latter option is more plausible. This is also supported by a previous study that showed that pwMS exhibit lower complexity during postural tasks [32,33]. Hence, using a predetermined state space template to fit pwMS’ walking data may serve to better highlight motor control impairments.

### Limitations

Several methodological decisions can affect the performance of the LDE to accurately classify pwMS. In this study, we focused on parameter selection for state space reconstruction. We did not, for example, focus on the algorithm selection or choosing between acceleration or angular velocity [34]. While no consensus has been documented, most studies have used the Rosenstein algorithm and linear acceleration data obtained with IMUs, both of which were employed in the present study. We acknowledge that stitched data from multiple laps in a long corridor, as in the present study, may not reveal the same dynamics of a system when assessed continuously. However, our approach has been previously used in pwMS and seems reasonable and feasible when used in clinical settings and with clinical populations, who may need close supervision during assessments as in, for example, a 6MWT [4,20,25]. Our study focused on RRMS with no evident gait impairments and low EDSS scores; classification of other MS phenotypes or subgroups, for example, of pwMS with pyramidal signs, was not explored. Although RRMS represents ~85% of cases, further studies should explore the use of the LDE to classify pwMS with different levels of disability and determine the generalizability of our findings across MS phenotypes. Significant between-group differences in BMI were found; however, to our knowledge, there is no evidence regarding the impact of BMI on stability during walking. Similarly, it is unknown whether other intrinsic factors related to MS such as fatigue and neuropsychological factors (e.g., mood) may affect motor performance, including stability. These factors could not be explored in the current dataset, as none of the participants reported fatigue and all had volunteered for the assessments conducted. Neuropsychological factors may be better examined in contexts where assessments using IMUs and relatively long walking tasks are part of routine evaluation.

## 5. Conclusions

The local divergence exponent (LDE) obtained using fixed parameters for state space reconstruction (*d_E_* = 5 and τ = 10) and the norm acceleration from a sternum-placed sensor, considering speed as a covariate, offered the best classification accuracy in pwMS with no evident gait impairments and at early stages of the disease. Our results provide further evidence towards achieving consensus for the use of the LDE in the monitoring of the disease progression and the development of potentially more sensitive outcome measures, which are greatly needed.

## Figures and Tables

**Table 1:** Median *d_E_* and τ calculated for each direction and for each sensor.

Sensor	Direction	Median *d_E_*	Range *d_E_*	Median τ	Range τ
Sternum (STR)	VT	7	[6, 8]	10	[6, 14]
	ML	7	[6, 9]	10	[4, 16]
	AP	7	[6, 10]	9	[4, 18]
	N	7	[6, 8]	10	[4, 14]
Lumbar (LUM)	VT	7	[5, 9]	7	[3, 13]
	ML	7	[6, 10]	5	[3, 10]
	AP	7	[6, 10]	5	[3, 13]
	N	7	[6, 9]	8	[3, 14]

VT: vertical, ML: mediolateral, AP: anteroposterior, N: norm.

**Table 2:** Participant demographics and clinical information.

Variable	HC (n = 23)	PwMS (n = 55)	*p*
EDSS (median) [IQR]	-	2.0 [1–2.5]	
Age (mean ± SD)	44.52 ± 12.13	41.69 ± 10.65	0.31
Sex ratio (m/f, % female)	8/15, (65%)	18/37, (67%)	0.86
Height (cm) (mean ± SD)	171.48 ± 29.03	170.54 ± 8.98	0.67
Body mass index (kg/m^2^) (mean ± SD)	23.96 ± 5.75	27.64 ± 4.98	<0.01

EDSS, Expanded Disability Status Scale; IQR, interquartile range; HC, healthy control; pwMS, people with multiple sclerosis. *p* values for between-group comparisons are presented on right.

**Table 3:** Classification accuracy (QDA) using LDE (short-term) and speed.

	Input	VT	ML	AP	N	3D *
Lumbar	Individual (I)	0.690	0.684	0.685	0.685	0.690
	Median (M)	0.701	0.703	0.694	0.721	0.679
	Fixed (F)	0.717	0.689	0.692	0.715	
Sternum	Individual (I)	0.685	0.678	0.704	0.705	0.770
	Median (M)	0.707	0.694	0.692	0.716	0.761
	Fixed (F)	0.775	0.779	0.771	**0.826**	

VT: vertical, ML: mediolateral, AP: anteroposterior, N: norm. Highest accuracy is highlighted in bold. * Indicates that for 3D data, I is *d_E_* = 3 and τ = 10, whereas M is *d_E_* = 5 and τ = 10.

## Data Availability

Data used and/or analyzed in this study are available from the corresponding author on reasonable request.

## Supplementary Materials

The following supporting information can be downloaded at: https://www.mdpi.com/article/10.3390/s25092819/s1, Table S1: Classification accuracy (QDA) using LDE short-term. Table S2. Classification accuracy (QDA) using LDE (short-term) + speed + age.
