# Supplementary material for: Multiple Sclerosis Classification Using the Local Divergence Exponent: Parameters Selection for State-Space Reconstruction
Source: Sensors (Basel). 2025 Apr 30;25(9):2819. doi: 10.3390/s25092819 (PMC12074368; doi:10.3390/s25092819)
Supplement: Supplementary file 1 [file sensors-25-02819-s001.zip › sensors-3550316-supplementary.pdf]

Supplementary Tables

Table S1. Classification accuracy (QDA) using LDE short-term.

	Input	VT	ML	AP	N	3D *
Lumbar	Individual (I)	0.704	0.690	0.702	0.700	0.679
	Median (M)	0.691	0.704	0.689	0.696	0.678
	Fixed (F)	0.688	0.697	0.689	0.689	-
Sternum	Individual (I)	0.707	0.706	0.708	0.696	0.664
	Median (M)	0.690	0.697	0.695	0.689	0.681
	Fixed (F)	0.682	0.766	0.709	0.673	-

VT: vertical, ML: mediolateral, AP: anteroposterior, N: norm. * Indicates that for 3D I is d_E =3 and τ=10, whereas M is d_E =5 and τ=10.

Table S2. Classification accuracy (QDA) using LDE (short-term) + speed + age.

	Input	VT	ML	AP	N	3D *
Lumbar	Individual (I)	0.715	0.738	0.714	0.711	0.718
	Median (M)	0.721	0.748	0.720	0.713	0.713
	Fixed (F)	0.721	0.729	0.719	0.711	-
Sternum	Individual (I)	0.710	0.725	0.722	0.710	0.709
	Median (M)	0.715	0.719	0.722	0.710	0.714
	Fixed (F)	0.714	0.734	0.719	0.711	-

VT: vertical, ML: mediolateral, AP: anteroposterior, N: norm. * Indicates that for 3D I is d_E =3 and τ=10, whereas M is d_E =5 and τ=10.
